# A Journey Undertaken by Families to Access General Surgical Care for their Children at Muhimbili National Hospital, Tanzania; Prospective Observational Cohort Study

**DOI:** 10.1007/s00268-022-06530-z

**Published:** 2022-04-12

**Authors:** Godfrey Sama Philipo, Zaitun Mohamed Bokhary, Neema Lala Bayyo, Soham Bandyopadhyay, Miriam Gerd Pueschel, Rajabu Athumani Bakari, Kokila Lakhoo

**Affiliations:** 1 Department of Epidemiology and Biostatistics, Muhimbili University of Health and Allied Sciences, P. O. Box 65001, Dar Es Salaam, Tanzania; 2 Faculty of Medicine, Branch of Global Surgical Care, The University of British Columbia, Vancouver, BC Canada; 3 Department of Surgery, Muhimbili National Hospital, Dar Es Salaam, Tanzania; 4 Department of Surgery, Muhimbili University of Health and Allied Sciences, Dar Es Salaam, Tanzania; 5 Nuffield Department of Surgical Sciences, University of Oxford, Oxford, UK

## Abstract

**Background:**

A majority of the 2 billion children lacking access to safe, timely and affordable surgical care reside in low-and middle-income countries. A barrier to tackling this issue is the paucity of information regarding children’s journey to surgical care. We aimed to explore children’s journeys and its implications on accessing general paediatric surgical care at Muhimbili National Hospital (MNH), a tertiary centre in Tanzania.

**Methods:**

A prospective observational cohort study was undertaken at MNH, recruiting patients undergoing elective and emergency surgeries. Data on socio-demographic, clinical, symptoms onset and 30-days post-operative were collected. Descriptive statistics and Mann–Whitney, Kruskal–Wallis and Fisher’s exact tests were used for data analysis.

**Result:**

We recruited 154 children with a median age of 36 months. The majority were referred from regional hospitals due to a lack of paediatric surgery expertise. The time taken to seeking care was significantly greater in those who self-referred (*p* = 0.0186). Of these participants, 68.4 and 31.1% were able to reach a referring health facility and MNH, respectively, within 2 h of deciding to seek care. Overall insurance coverage was 75.32%. The median out of pocket expenditure for receiving care was $69.00. The incidence of surgical site infection was 10.2%, and only 2 patients died.

**Conclusion:**

Although there have been significant efforts to improve access to safe, timely and affordable surgical care, there is still a need to strengthen children’s surgical care system. Investing in regional hospitals may be an effective approach to improve access to children surgical care.

## Introduction

Access to health care is one of the most basic human rights supported by the Universal Declaration of Human Rights [1]. Indeed, the 2030 Agenda for Sustainable Development, approved by the United Nations in 2015, includes the key health-related target (Sustainable Development Goal 3.8) of universal health coverage (UHC) [2]. However, more than 94% of the global population lacking access to safe, timely and affordable surgical are from low-and middle-income countries (LMICs), and majority are children [3]. Approximately, 88 million individuals incurred catastrophic expenditures from seeking surgical and anaesthesia care [3]. Children represent the majority of the population in Tanzania and other LMICs [4, 5]. Failure to meet surgical needs to children may be a barrier to achieving the UHC and advancing the human rights agenda [6].

There is paucity of data, especially in LMICs, on access to children’s surgical care and related contextual challenges [7–10]. Over 70% of Tanzanians reside in rural setting and are served by district hospitals [11]. The ongoing developments to ensure access to essential surgical care even in district hospitals may not always address surgical needs for children. In Tanzania, Muhimbili National Hospital (MNH) is one of the centres in the country with developed capacity to provide children surgical care – in terms of available workforce and infrastructure – but is considerably far from some places where it receives referrals. Inadequate data on patients’ journeys to access surgical care limit strategic design and implementation of policies for improvement [12]. Much of the data currently being used are from the Global North, where the situation is vastly different, hence may fail to reflect and realise paediatric surgical care needs in Tanzania [13]. This study, therefore, aimed to assess the journey patients make to receiving general paediatric surgical care at MNH and explore other Lancet Commission on Global Surgery (LCoGS) indicators related to safety and cost burden of healthcare.

## Methodology

A prospective observational cohort study was undertaken from 2019 to 2020 at MNH in Dar Es Salaam (Coastal region of Tanzania). This is a tertiary national referral hospital capable of providing care for complex surgical conditions, receiving diversity of paediatric surgery patients from all over the country. The centre has two paediatric operating theatres rooms and a 60-bed paediatric surgery ward.

We randomly included patients undergoing elective or non-elective general surgery at MNH, aged 11-year-old or younger, and whose parent/caregiver consented on their behalf for participation and follow-up. We excluded those needing cardiac, trauma, neuro and plastic surgery as they are treated in separate respective institutes or units of MNH. Participation was voluntary and did not impact or change the care that they were receiving.

Collection of demographics, clinical and follow-up data was done by two study coordinators, both medical doctors and registrars at the department of paediatric surgery with a minimum of 2 years of experience. They had undergone the necessary research governance and ethics training for data collection. A Swahili structured questionnaire was used to interview and collect information from the parent/caregiver from the onset of the child’s symptoms to 30-days post-operatively. This included participant and caregiver demographics, time to seeking, reaching and receiving surgical care, referral pattern, mode of transportation, insurance status and dates of admission, surgery and discharge/death. Distances travelled (km) from home to a referring health facility and/or to MNH were estimated by using Google Maps (https://www.google.com/maps): a free online tool which has been reported to be an accurate way of assessing distances [14, 15]. We used Clavien-Dindo system for grading adverse events (i.e. complications) which occur because of surgical procedures [16]. Out-of-pocket (OOP) expenditure incurred by the patient’s family on their journey to receiving care were collected in Tanzanian Shillings (TZS) and converted to US dollars (USD), a conversion rate of as of 12/07/2021 ($1 = 2319 TZS). A case report form (CRF) was used to collect data on patients’ clinical information and outcomes, and patients were followed-up for 30 days while in the ward, or by phone and/or during clinic visits after discharge. Anonymous data were collected and stored in a secure REDCap database hosted by MNH that was accessible only to researchers.

Data were described in proportions for categorical variables, and medians and interquartile ranges (IQR) for quantitative variables. The Mann–Whitney Test and Kruskal–Wallis Test were used to determine differences between sub-groups where the explanatory variable was categorical, and the response variable was quantitative while Fisher’s exact test for differences between sub-groups where both explanatory and response variables were categorical. A multiple linear regression was calculated to predict time taken for a patient to present at MNH based on the distance of their home and referring centre from MNH. Data were analysed using Stata 15.1. We used the Strengthening the Reporting of Observational Studies in Epidemiology (STROBE) statement for observational studies to report our findings [17]. Ethical approval was received from the MNH Institutional Review Board (IRB No: MNH/IRB/2019/036).

## Results

### Demographics

**Fig. 1:**
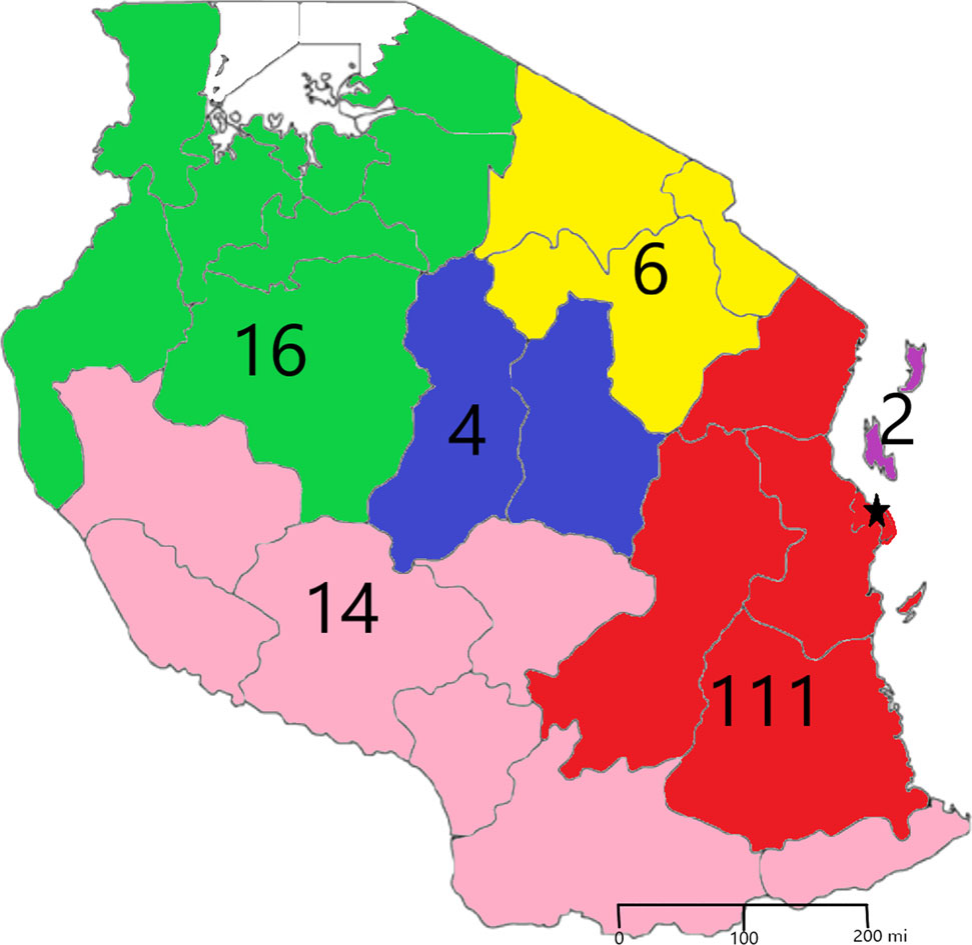
Zones participants reside in. Central Zone (blue) = 4 participants. Northern Zone (yellow) = 6 participants. Coastal Zone (red) = 111 participants. Southern Zone (pink) = 14 participants. Green Zone (green) = 16 participants. Zanzibar (purple) = 2 participants. Unknown = 1 participant. The black star represents the location of Muhimbili National Hospital

**Fig. 2:**
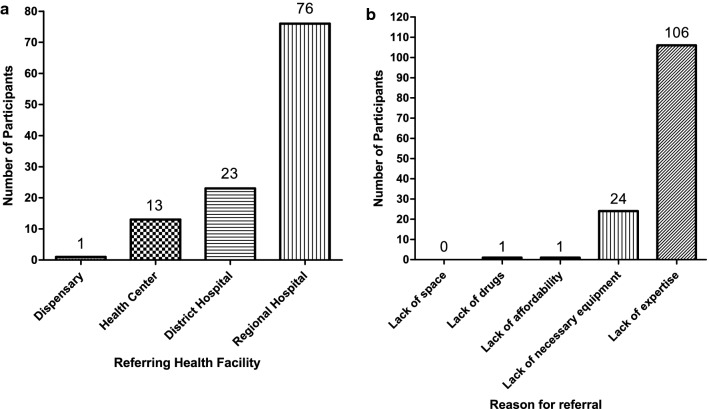
**a** Referring health facilities and **b** the main reasons for referral of children seeking surgical care at MNH

A total of 154 children with a median age at admission of 36 (IQR: 18 – 56) months participated in this study. The majority were from Coastal zone (n = 109/154, 71.2%) (Fig. 1) and male (n = 99/154, 64.3%) (Table 1). *Appendix S1* details the regions-as per political divisions—the children resided in [18]. Most participants (n = 120/154, 77.9%) were referred from another health facility. Of these, 63.3% (n = 76/120) were from regional hospitals, with lack of paediatric surgery expertise being the main reason for referral (n = 106/120, 88.3%) (Fig. 1). 141 patients (92.2%) underwent elective surgery; (Fig. 2) anorectal malformation being the most common diagnosis (38/154, 24.7%). Most children had only 2 adults in their immediate family (n = 110/154, 71.4%).

**Table 1:** Sociodemographic characteristics

Patient Demographics		Frequency (n)	Percentage (%)
Gender	Female	53	34.4
	Male	99	64.3
	Ambiguous	2	1.3
Referral Status	Self-referral	120	77.9
	Referred from health facility	34	22.1
Diagnoses	Anorectal Malformation	38	24.7
	Hirschsprung Disease	22	14.3
	Urogenital anomalies	32	20.8
	Appendicitis	6	3.9
	Hernia	20	13.0
	Biliary atresia	3	1.9
	Duodenal stenosis	6	3.9
	Abdominal injuries	2	1.3
	Nephroblastoma	10	6.5
	Lipoma	6	3.9
	Hypersplenism	6	3.9
	Intussusception	3	1.9
Type of surgery	Elective surgery	141	92.2
	Emergency surgery	12	7.8
Caregiver Demographics		Frequency (n)	Percentage (%)
Occupation	Self employed	98	63.6
	Public employee	24	15.6
	Homemaker	28	18.2
	Unemployed	4	2.6
Education	Higher Education	25	16.2
	No education	11	7.1
	Primary	63	40.9
	Secondary	55	35.7
Age group	15-19	3	2.0
	20-24	16	10.4
	25-29	39	25.3
	30-34	40	26.0
	35-39	31	20.1
	40-44	17	11.0
	>45	8	5.2
Marital status	Married/cohabiting	125	81.2
	Single	18	11.7
	Widowed/Separated	10	6.5
	Unknown	1	0.7
Other members of the family		Widowed/Separated	IQR (25, 75 percentiles)
Adults in immediate family		Unknown	2 – 2
Children in immediate family		2	2 – 4

### Time to Seek Care, Reach Care and Receive Care

**Fig. 3:**
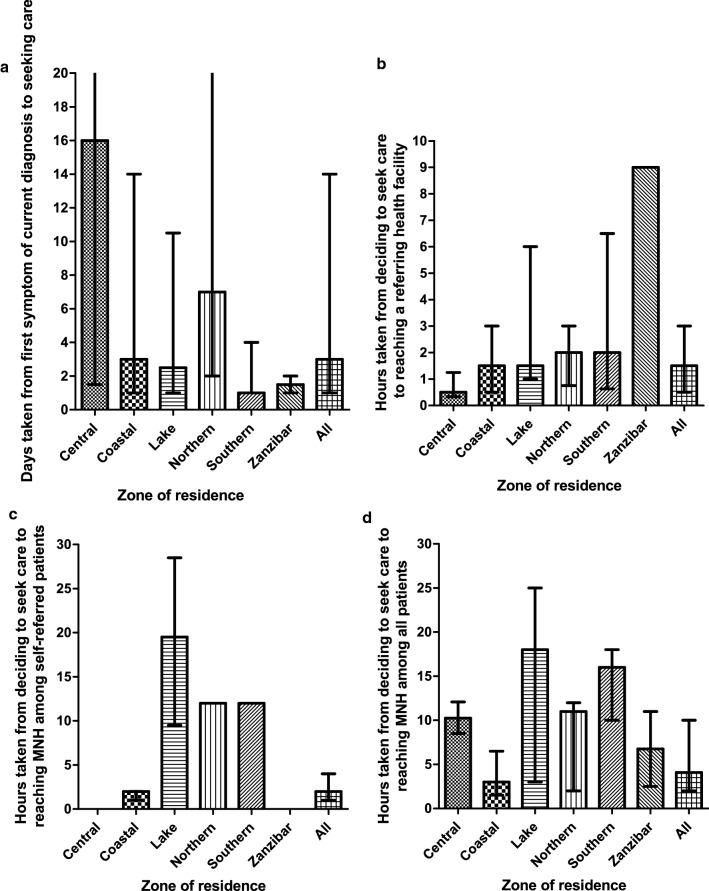
**a** Days taken from first symptom of current diagnosis to seeking care **b** Hours taken from deciding to seek care to reaching a referring health facility **c** Hours taken from deciding to seek care to reaching MNH among self-referred patients **d** Hours taken from deciding to seek care to reaching MNH among all patients. The results are segregated based on the participant’s zone of residence. The height of the bars represents the median, and the error bars represent the interquartile range

Participants took a median time of 3 days (IQR: 1 – 14) to seek care from their first symptom; this varied by the zone of the patient (Fig. 3 and *Appendix S2*). From deciding to seek care, it took participants 1.5 h (0.5 – 3) and 4.08 h (IQR: 2 – 10) on average to reach a referring health facility and MNH for care, respectively. Only 31.1% (46/148) of participants were able to reach MNH-compared to 68.4% (80/117) who reached a referring health facility-within 2 h of deciding to seek care (*Appendix S3*). Travel to a referring health facility could involve rough roads and/or tarmac roads. 13 patients had to travel on rough roads, where they spent a median time of 2 h (IQR: 1.5 – 2). The medial time spent on tarmac roads was 1 h (IQR: 0.5 – 3).

**Fig. 4:**
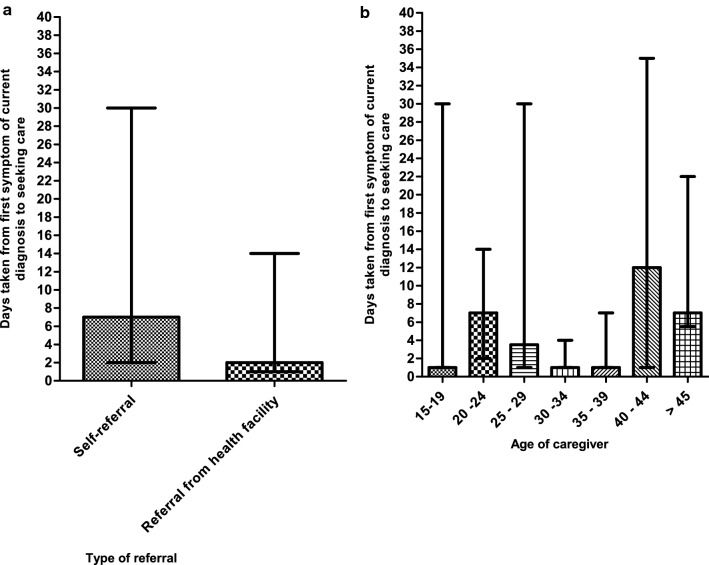
The days taken to seek care from first symptom of current diagnosis based on (**a**) the type of referral, and (**b**) the age of the caregiver. The height of the bars represents the median, and the error bars represent the interquartile range

**Fig. 5:**
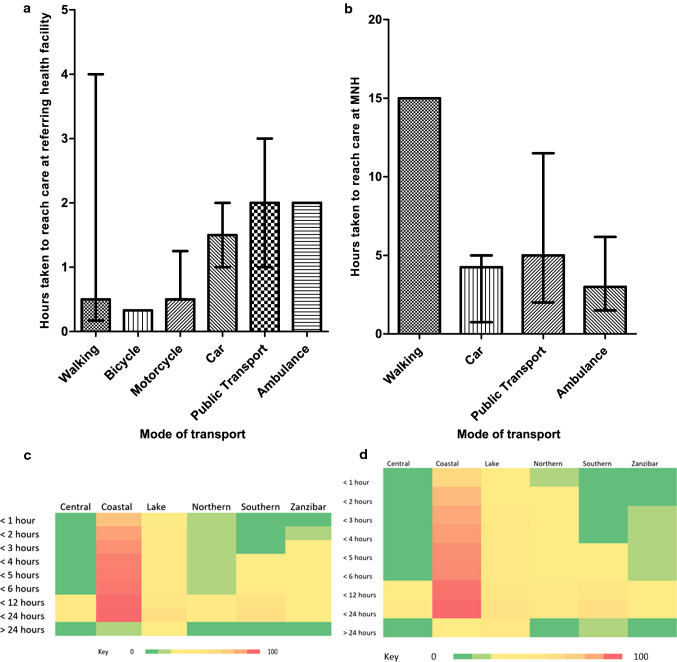
The relationship between mode of transport and (**a**) the hours taken from deciding to seek care to reaching a referring health facility, and (**b**) the hours taken to reaching care at MNH among all patients. The height of the bars represents the median, and the error bars represent the interquartile range. (**c**) Heat map of the hours taken from deciding to seek care to reaching MNH among all patients taken. MNH: Muhmbili National Hospital. (**d**) Heat map of the hours taken from deciding to seek care to reaching a referring health facility

A significant regression equation was found (F (2, 113) = 94.96, *p* < 0.0001), with an R2 of 0.627. The predicted time (hours) taken is equal to *1.061* + *0.003 (distance in km of their home from MNH)* + *0.018 (distance of their referring health facility from MNH)*. Time taken to present to MNH increased by 0.003 h for each km their home was from MNH (*p* = 0.070) and 0.018 h for each km their referring health facility was from MNH (*p* < 0.001). The time taken from first symptom of current diagnosis to seeking care was significantly greater in those who self-referred (median: 7 days; IQR: 2 – 30) compared to those referred through a health facility (median: 2 days; IQR: 1 – 14) (*p* = 0.0186). A significant difference was also noted in the time to seeking care between different caregivers’ age groups (*p* = 0.0112) (Fig. 4 and *Appendix S4*). The time taken to reaching care at a referring health facility was significantly greater in those who did not travel by a motorcycle (median: 1.5 h; IQR: 0.75 – 3) compared to those who did (median: 0.5 h; IQR: 0.5 – 1.25) (*p* = 0.0048) and in those who used public transport (median: 2 h; IQR: 1 – 3) compared to those who did not (median: 1 h; IQR: 0.5 – 2) (*p* = 0.0004) (Fig. 5 and *Appendix S4*). The time taken to reach care at MNH was significantly greater in those who used public transport (median: 5 h; IQR: 2 – 11.5) compared to those who did not (median: 3 h; IQR: 1.5 – 5) (*p* = 0.0280) (Fig. 5 and *Appendix S4*). *Appendix S5 and S6* show the original location of patients who were able to present to MNH within 2 h of deciding to seek care and the factors affecting time taken to access care.

The majority of participants were referred to MNH for elective surgery (n = 141/153, 92.2%) and had a pre-operative ASA score of 1 (n = 137/153, 89.5%). The incidence of post–operative SSI was 10.2% (15/147) (Table 2). The time taken from first symptom of current diagnosis to seeking care from a healthcare provider was significantly shorter in those who had emergency surgery (*p* = 0.0198) (elective surgery – median: 3 (IQR: 1 – 14) days; emergency surgery – median: 1 (IQR: 1 – 4) day. There was no significant difference in the time taken to reach care at a referring health facility between those who had emergency surgery and elective surgery (*p* = 0.4361) (elective surgery – median: 1.5, IQR: 0.5—3 h; emergency surgery – median: 1.25, IQR: 0.5 – 2 h). The time taken to present at MNH was significantly shorter in those who had emergency surgery (*p* = 0.0396) (elective surgery – median: 4.25, IQR: 2 – 11 h; emergency surgery – median: 2.75, IQR: 1 – 5 h). The median time from admission to receiving surgical care was 3 (IQR: 1 – 14) days; all emergency surgery was conducted within a day.

**Table 2:** Operative details and comparison of children underwent elective and emergency surgeries

Variable		Elective (n = %)	Emergency (n = %)	*P* value
ASA Score	1	126 (89.4)	11 (91.7)	0.999
	2	9 (6.4)	0 (0.0)	
	3	1 (0.7)	0 (0.0)	
	Unknown	5 (3.6)	1 (8.3)	
Post-Op Complication	Mild	6 (4.3)	1 (8.3)	0.024
	Moderate	2 (1.4)	2 (16.7)	
	No complication	133 (94.3)	9 (75.0)	
Clavien Dindo	I	63 (44.7)	0 (0.0)	0.002
	II	76 (53.9)	12 (100.0)	
	III	1 (0.7)	0 (0.0)	
	IV	0 (0.0)	0 (0.0)	
	Unknown	1 (0.7)	0 (0.0)	
Surgical Site Infection	No	124 (87.9)	8 (66.7)	0.022
	Yes	11 (7.8)	4 (33.3)	
	Unknown	6 (4.3)	0 (0.0)	
Discharge after surgery	Recovery room then ward	112 (79.4)	10 (83.3)	0.320
	Intensive care	29 (20.6)	1 (8.3)	
	Unknown	0 (0.0)	1 (8.3)	
Hospitalization status at 30 days	Alive and discharged	113 (80.1)	10 (83.3)	0.999
	Alive still in ward	20 (14.2)	2 (16.7)	
	Dead	2 (1.4)	0 (0.0)	
	Unknown	6 (4.3)	0 (0.0)	

There was a significant difference in post-operative complications in those who underwent elective surgery compared to emergency surgery (*p* = 0.002). A greater proportion of children had mild (elective: n = 6/141, 4.3%; emergency: n = 1/12, 8.3%) and moderate (elective: n = 2/141, 1.4%; emergency: n = 2/12, 16.7%) post-operative complications in the emergency surgery sub-group. Individuals were significantly more likely to have a post-operative surgical site infection (SSI) if they underwent emergency surgery (*p* = 0.022) (Table 2).

### Insurance Status

Most participants had insurance (n = 116/154, 75.32%). Children were significantly more likely to have insurance if they were undergoing elective surgery (n = 111/141, 78.7%) over emergency surgery (n = 4/12, 33.3%) (*p* = 0.002). The median total out of pocket expenditure for receiving care at both referring health facility and MNH was $69.00 (Table 3). This was significantly greater among those referred through another health facility compared to those who self-referred to MNH (*p* = 0.0135). There was weak evidence that out-of-pocket expenditure was greater in those who did not have insurance (*p* = 0.0755) (insurance – median: $60.37, IQR: $17.90– $155.24; no insurance – median: $97.02, (IQR: $40.97– $232.86). Table 4 shows the relationship between self-reported socio-economic status and the various factors identified above to be significantly related to timely surgical access.

**Table 3:** Assets owned, financial status, insurance status and expenditures of participants

Variable	Frequency (n)	Percentage (%)
Materials owned by caregiver [%]	Land: 97 House: 92 Animals: 48 Bank account: 59 Electrical equipment: 114 Bicycle: 35 Motor vehicle: 32	Land: 63.0 House: 59.7 Animals: 31.2 Bank account: 38.3 Electrical equipment: 74.0 Bicycle: 22.7 Motor vehicle: 20.8
Self-reported socioeconomic status: amount of money owned by caregiver [%]	Enough money for food: 47 Enough money for food and clothes only: 54 Enough money for food, clothes, and savings: 49 Enough money for the above and certain expensive goods: 4	Enough money for food: 30.5 Enough money for food and clothes only: 35.1 Enough money for food, clothes, and savings: 31.8 Enough money for the above and certain expensive goods: 2.6
Insurance status	National Health Insurance Fund: 114 Private Insurance: 2 No insurance: 38	National Health Insurance Fund: 74.0 Private Insurance: 1.3 No insurance: 24.7

Expenditure	Median (USD)	IQR (25, 75 percentiles)
Expenditure on food per day	$4.31	$2.59 – $4.31
Out of pocket expenditure for travelling to the referring health facility	$1.29	$0.43 – $4.31
Out of pocket expenditure for travelling to MNH	$4.31	$0.69 – $10.78
Out of pocket expenditure for care at referring health facility	$28.03	$6.47 – $86.24
Out of pocket expenditure for care at MNH	$20.27	$4.48 – $64.68
Out of pocket expenditure of receiving care at both referring health facility and MNH	$69.00	$2.16 – $172.49

**Table 4:** Relationship between self-reported socioeconomic status and potential factors related to timely surgical access. MNH: Muhimbili National Hospital

		Self-reported socioeconomic status				*p*-value
		Enough money for food (n = 47)	Enough money for food and clothes only n = 54)	Enough money for food, clothes, and savings (n = 49)	Enough money for the food, clothes, savings and certain expensive goods (n = 4)	
Urgency of surgery n (%)	Elective	41 (87.2)	50 (92.6)	47 (95.9)	3 (75.0)	0.283^a^
	Emergency	5 (10.6)	4 (7.4)	2 (4.1)	1 (25.0)	
	Missing	1 (2.1)	0 (0.0)	0 (0.0)	0 (0.0)	–
Zone of residence of participants n (%)	Central	0 (0.0)	3 (5.6)	1 (2.0)	0 (0.0)	0.617^a^
	Coastal	33 (70.2)	42 (77.8)	32 (65.3)	4 (100.0)	
	Lake	5 (10.6)	2 (3.7)	9 (18.4)	0 (0.0)	
	Northern	2 (4.3)	2 (3.7)	2 (4.1)	0 (0.0)	
	Southern	5 (10.6)	5 (9.3)	4 (8.2)	0 (0.0)	
	Zanzibar	1 (2.1)	0 (0.0)	1 (2.0)	0 (0.0)	
	Missing	1 (2.1)	0 (0.0)	0 (0.0)	0 (0.0)	–
Absolute distance travelled from home to MNH in km Median (IQR)		46 (16 – 548)	32 (18 – 449)	23 (16 – 286)	19 (12.6 – 232.5)	0.497^b^
Referral Status	Self-referral	37 (78.7)	49 (90.8)	32 (65.3)	2 (50.0)	0.006^a^
	Referred from health facility	19 (21.3)	5 (9.3)	17 (34.7)	2 (50.0)	
Caregiver age group	15–19	0 (0.0)	2 (3.7)	1 (2.0)	0 (0.0)	0.375^a^
	20–24	5 (10.6)	7 (13.0)	4 (8.2)	0 (0.0)	
	25–29	12 (25.5)	16 (29.6)	11 (22.5)	0 (0.0)	
	30–34	8 (17.0)	11 (20.4)	18 (36.7)	3 (75.0)	
	35–39	14 (29.8)	8 (14.8)	9 (18.4)	0 (0.0)	
	40–44	4 (8.5)	7 (13.0)	5 (10.2)	1 (25.0)	
	> 45	4 (8.5)	3 (5.6)	1 (2.0)	0 (0.0)	
Mode of transport taken to reaching care at MNH	Walking	2 (4.3)	2 (3.7)	1 (2.0)	0 (0.0)	0.269^a^
	Bicycle	0 (0.0)	1 (1.9)	0 (0.0)	0 (0.0)	
	Motorcycle	3 (6.4)	5 (9.3)	4 (8.2)	0 (0.0)	
	Car	3 (6.4)	4 (7.4)	6 (12.2)	2 (50.0)	
	Public Transport	27 (57.5)	34 (63.0)	21 (42.9)	0 (0.0)	
	Ambulance	1 (2.1)	0 (0.0)	0 (0.0)	0 (0.0)	
	Missing	11 (23.4)	8 (14.8)	17 (34.7)	2 (50.0)	–

^*a*^ = *Fisher’s exact test. *^*b*^ = *Kruskal–Wallis test*

## Discussion

### Key Findings

Patients travel long distances, navigate a complicated referral system, and incur significant costs in seeking and receiving paediatric surgical care. More than two-thirds of children saw a healthcare provider at a referring health facility within 2 h, but approximately a third of all children reached a tertiary hospital (MNH) within 2 h of deciding to seek care. This is still in stark contrast to other low-resource settings, where approximately four-fifths of the population are unable to access surgical care within 2 h [19]. For those who required emergency procedures, 50% reached MNH within 2 h of deciding to seek care and were more likely to have post-operative complications.

### Recommendations

Lack of paediatric surgery expertise was the main reason that 77.9% patients were referred from other healthcare facilities. This is a reversal of the 2008 findings that self-referrals accounted for 72.5% of presentations at MNH for both surgical and non-surgical conditions [20]. Self-referral is thought to be associated with later presentation when the disease is more severe and worse prognosis. In our study, self-referring was associated with an increased time to seeking care. Similar findings have been reported in previous studies: a study in Uganda reported although 90% of participants were identified by family members to be suffering from an illness, only 14% sought medical attention immediately [21].

Existing benchmarks define paediatric surgical procedures that can be provided at various levels of healthcare based on resources, and these guide effective referral [22]. Based on these benchmarks, the majority of the referrals in our study needed tertiary level care. However, about 20.8% (32/154) of children had conditions (hernias, appendicitis and lipoma) which could have been treated at lower-level hospitals. Increased burden of managing these cases in tertiary hospitals may limit surgical care provision for both complex and common conditions [23]. If adequate resources are available, regional hospitals become the cornerstone of LMICs surgical care [23–25].

In addition to infrastructure developments, training by local and international providers need to be prioritized [26]. This can be achieved by training multidisciplinary teams of children surgical providers [27] as well as including task-shifting and sharing [28, 29]. Defining regional hospitals as centres for providing paediatric surgery and incorporating telemedicine may leapfrog physical barriers and surgical specialist shortages. This will ensure timely access to surgical care, reduce the number of preventable referrals and overcrowding at higher-level hospitals [30]. Unit costs and the relative shares of capital costs are generally lower at primary-level hospital [31]. Effective treatment depends on all steps of a healthcare system working harmoniously, from timely seeking and reaching healthcare, appropriate triage for surgery or referral, to proper transportation for care in an adequately resourced facility for better outcomes [32]. The modern concepts of improving value in healthcare emphasize the importance of considering value across the whole patient pathway from symptoms to care and rehabilitation [33]. It was shown that surgical outcomes will remain poor in Africa unless perioperative care is improved [24]. This include the pathway to care, which is a critical and the most challenging period that may determine treatment outcomes.

Although there was a higher overall health insurance coverage (75%), those who were undergoing emergency surgery had 33.3%, which is comparable to findings of another study done on surgical patients in Northern Tanzania (45.5%) and to Tanzania’s general population (32% in 2019) [34, 35]. Patients are likely to have received their health insurance after being planned for elective surgery. This may explain the considerable out of pocket expenses among our study participants, related to both medical and non-medical expenses, and weak evidence that having health insurance protected patients from significant out of pocket expenditures. It was higher in those who were referred through another health facility. About 76.8% of Tanzanian are living below the poverty line ($3.20 per day) [36]: 65.8% and 85.5% are estimated to be at risk of catastrophic and impoverishing expenditures from seeking surgical care, well above the target of 0% by 2030 [3, 25]. It is important that paediatric surgical care is also financially accessible [37]. A median out-of-pocket expenditure of $69 for receiving surgical care in this study was considerably higher than the cost incurred for paediatric inpatient care in district hospitals in Kenya ($14.1) and Tanzania ($5.5) [37, 38]. The consequences of out-of-pocket expenditure are pushing individuals and households into poverty, most of these are in rural settings of Tanzania and other LMICs. In these settings, $69 can equate to a month’s salary for many, forming a barrier to individuals seeking care [39, 40]. An argument for deductibles and co-payments is to reduce the moral hazard of patients, but it is highly unlikely that children are considering the cost of their care when they are asking for it [41, 42]. Therefore, one method of reducing the cost burden on caregivers would be a policy of free inpatient care for all children. However, a large proportion of costs in hospitals in Tanzania is related to food [38]. MNH has a policy of free provision of food for children coming from far. Activity-based costing may be adopted and utilised in Tanzania and similar settings to reduce costs of hospital food and other direct nonmedical costs [43].

### Limitations

A limitation of this study is that it is based on patients who presented to MNH; we are unable to ascertain the treatment pathways for those who did not get to MNH. Future studies should consider understanding pathways to the regional hospitals. Furthermore, future studies should use qualitative methods to explore patient experiences in seeking and receiving surgical care.

## Conclusion

This is the first report on whether paediatric patients in Tanzania have access to safe, timely and affordable surgical care. The majority of patients are able to access paediatric surgical care at referring health facilities within 2 h, especially those who need emergency surgery. There is a low rate of post-operative complications after paediatric surgery in Tanzania. However, paediatric surgery leads to considerable out of pocket expenses. Whilst great strides have been made by the Tanzanian government and various external partners to strengthen the surgical system in Tanzania, there now needs to be a greater focus on policies for paediatric patients. Indeed, efforts to scale up surgical care in in Tanzania and other LMICs should consider the needs of paediatric patients.

## Appendix

See Tables 5,

**Table 5:** Regions of residence of participants

Zone	Region	Frequency (% of zone)
Central	Dodoma	4 (100.0)
Coastal	Dar Es Salaam	76 (69.7)
	Iringa	1 (0.9)
	Mbeya	1 (0.9)
	Morogoro	12 (10.8)
	Pwani	11 (9.9)
	Tanga	9 (8.1)
	Zanzibar	1 (0.9)
Lake	Geita	2 (12.5)
	Kagera	1 (6.3)
	Kigoma	1 (6.3)
	Mara	6 (37.5)
	Mwanza	2 (12.5)
	Tabora	4 (25.0)
Northern	Arusha	2 (33.3)
	Kilimanjaro	4 (66.7)
Southern	Iringa	2 (14.3)
	Lindi	4 (28.6)
	Mbeya	3 (21.4)
	Mtwara	3 (21.4)
	Njombe	1 (7.1)
	Ruvuma	1 (7.1)
Zanzibar	Pemba	1 (50.0)
	Zanzibar	1 (50.0)

6,

**Table 6:** Duration and distance travelled by participants to reach care based on their zone of residence. MNH: Muhimbili National Hospital

	Central, median (IQR)	Coastal, median (IQR)	Lake, median (IQR)	Northern, median (IQR)	Southern, median (IQR)	Zanzibar, median (IQR)	All zones, median (IQR)
Duration							
Days taken from first symptom of current diagnosis to seeking care	16 (1.5 – 555)	3 (1 – 14)	2.5 (1 – 10.5)	7 (2 – 365)	1 (1 – 4)	1.5 (1 – 2)	3 (1 – 14)
Hours taken from deciding to seek care to reaching a referring health facility	0.5 (0.33 – 1.25)	1.5 (0.5 – 3)	1.5 (1 – 6)	2 (0.75 – 3)	2 (0.625 – 6.5)	9 (9 – 9)	1.5 (0.5 – 3)
Hours taken from deciding to seek care to reaching MNH among self-referred patients	NA	2 (1 – 2)	19.5 (9.5 – 28.5)	12 (12 – 12)	12 (12 – 12)	NA	2 (1 – 4)
Hours taken from deciding to seek care to reaching MNH among all patients	10.25 (8.5 – 12.08)	3 (1.5 – 6.5)	18 (3 – 25)	11 (2 – 12)	16 (10 – 18)	6.75 (2.5 – 11)	4.08 (2 – 10)
Distance Travelled (km)							
Absolute distance travelled from home to MNH	215.5 (50.5 – 393)	23 (16 – 443)	109 (20 – 829)	103.15 (12 – 286)	193 (22 – 321)	179.5 (22 – 337)	30 (16 – 443)
Absolute distance from home to referring health facility	13.8 (6.35 – 96.35)	12.5 (5.5 24.95)	263.8 (82.8 – 826.6)	281.35 (89.95 – 524.75)	34.4 (6.9 – 65)	131 (131 – 131)	15.2 (5.7 – 77)
Absolute distance from referring health facility to MNH	448.6 (448.6 – 448.6)	8.1 (6.6 – 76.35)	1130.6 (7.85 – 1131.55)	298.75 (27.85 – 549.9)	560.6 (454.1 – 822.9)	86.4 (86.4 – 86.4)	10.65 (6.6 – 338.1)
Distance travelled to MNH (self-referred)	NA	66 (16 – 563)	20 (15.5 – 193)	97.75 (4.5 – 191)	257 (193 – 321)	337 (337 – 337)	109 (16 – 443)
Distance travelled to MNH (referred from health facility)	462.4 (454.95 – 544.95)	29.2 (15.1 – 189.1)	1192.75 (117.5 – 1394.4)	612.35 (541.15 – 651.3)	603.3 (456.9 – 883.2)	217.4 (217.4 – 217.4)	45.05 (20.5 – 502.7)

7,

**Table 7:** Original location of patients who were able to present to MNH within 2 h of deciding to seek care

Type of surgery	Referral Type	Level of the referring hospital	Zones	Region	District	Village	Name of the referring hospital
Emergency	Self-referral		Coastal	Dar Es salaam	Ilala	Kariakoo	
Emergency	Healthcare provider referral		Coastal	Dar Es salaam	Temeke	Temeke	Regency hospital
Emergency	Healthcare provider referral	Dispensary	Coastal	Dar Es salaam	Kinondoni	Kinondoni	Dr Amir dispensary
Emergency	Healthcare provider referral	District hospital	Coastal	Dar Es salaam	Ilala	Gongo la mboto	Amana Regional refferal hospital
Emergency	Healthcare provider referral		Coastal	Dar Es salaam	Kinondoni	Kigamboni	
Emergency	Healthcare provider referral	Health center	Northern	Arusha	Arumeru	Poli	Temeke hospital
Elective	Self-referral		Coastal	Dar Es salaam	Ubungo	Ubungo	
Elective	Self-referral		Coastal	Dar Es salaam	Kinondoni	Kinondoni	
Elective	Healthcare provider referral	Regional hospital	Coastal	Dar Es salaam	Kinondoni	Mwananyamala	Mwananyamala regional refferal hospital
Elective	Self-referral		Coastal	Dar Es salaam	Temeke	Mbagala	
Elective	Self-referral		Coastal	Dar Es salaam	Kinondoni	Kijitonyama	
Elective	Healthcare provider referral	Regional hospital	Coastal	Dar Es salaam	Ilala	Ilala	Mwananyamala regional refferal hospital
Elective	Healthcare provider referral	Regional hospital	Coastal	Dar Es salaam	Kinondoni	Mikocheni	Mwananyamala referral hospital
Elective	Healthcare provider referral	Regional hospital	Coastal	Dar Es salaam	Temeke	Mbagala	Temeke regional referral hospital
Elective	Self-referral		Coastal	Dar Es salaam	Temeke	Yombo	
Elective	Healthcare provider referral	Regional hospital	Coastal	Dar Es salaam	Kinondoni	Magomeni	Mwananyamala regional refferal hospital
Elective	Self-referral		Coastal	Dar Es salaam	Temeke	Mbagala	
Elective	Self-referral		Coastal	Dar Es salaam	Ilala	VIngunguti	
Elective	Healthcare provider referral	Regional hospital	Coastal	Dar Es salaam	Ilala	Kiwalani	Amana regional refferal hospital
Elective	Self-referral		Coastal	Pwani	Kibaha	Kibaha	
Elective	Self-referral		Coastal	Pwani	Mkuranga	Mwanambaya	
Elective	Healthcare provider referral	Regional hospital	Coastal	Dar Es salaam	Ilala	Ukonga	Amana regional refferal hospital
Elective	Healthcare provider referral		Coastal	Dar Es salaam	Ilala	Gongo la mboto	Dr Amir hospital
Elective	Healthcare provider referral	Regional hospital	Coastal	Dar Es salaam	Ilala	kigogo	Mwananyamala reginal refferal hospital
Elective	Healthcare provider referral	Regional hospital	Coastal	Dar Es salaam	Ilala	Mchikichini	Amana hospital
Elective	Healthcare provider referral	District hospital	Coastal	Dar Es salaam	Ilala	Kinyerezi	Amana regional refferal hospital
Elective	Healthcare provider referral	Regional hospital	Coastal	Dar Es salaam	Ubungo	Mabibo	Amana regional refferal hospital
Elective	Healthcare provider referral	District hospital	Coastal	Dar Es salaam	Kinondoni	Mikocheni	Sinza hospital
Elective	Healthcare provider referral	Regional hospital	Coastal	Dar Es salaam	Kinondoni	Mwananyamala	Mwananyamala regional refferal hospital
Elective	Healthcare provider referral	Regional hospital	Coastal	Iringa	Kilolo	Boma la ng'ombe	Mwananyamala hospital
Elective	Healthcare provider referral	Health center	Coastal	Pwani	Kibaha	Kibaha	Tumbi hospital
Elective	Healthcare provider referral	Regional hospital	Coastal	Dar Es salaam	Temeke	Mbagala	Temeke hospital
Elective	Self-referral		Coastal	Dar Es salaam	Kigamboni	Ujindoni	
Elective	Self-referral		Coastal	Dar Es salaam	Ilala	Chanika	
Elective	Healthcare provider referral	Regional hospital	Coastal	Dar Es salaam	Kinondoni	Tegeta	Mwananyamala regional referral hospital
Elective	Self-referral		Coastal	Dar Es salaam	Ilala	Tabora	
Elective	Self-referral		Coastal	Dar Es salaam	Ubungo	Kibamba	
Elective	Healthcare provider referral		Coastal	Dar Es salaam	Kinondoni	Bunju	Lugalo hospital
Elective	Self-referral		Coastal	Dar Es salaam	Kigamboni	Gezaulole	
Elective	Healthcare provider referral	District hospital	Coastal	Dar Es salaam	Ilala	Pugu	Amana regional refferal hospital
Elective	Self-referral		Coastal	Dar Es salaam	Ilala	Ilala	
Elective	Self-referral		Coastal	Dar Es salaam	Kigamboni	Kigamboni	
Elective	Healthcare provider referral	Regional hospital	Lake	Geita	Geita	Geita	Mwananyamala regional refferal hospital
Elective	Self-referral		Lake	Mara	Serengeti	Sedeko	
Elective	Healthcare provider referral	Regional hospital	Northern	Kilimanjaro	Same	Hedaru	Mkuranga hospital
Elective	Self-referral						

8

**Table 8:** Factors affecting time taken to access care

		A) Days taken from first symptom of current diagnosis to seeking care, median (IQR)	B) Hours taken to reach care at referring health facility, median (IQR)	C)Hours taken to reaching care at MNH (self-referred patients) (IQR)	D) Hours taken to reaching care at MNH among all patients, median (IQR)
*Type of referral*					
Referral from health facility	Dispensary	5 (5 – 5)	0.5 (0.5 – 0.5)	NA	1 (1 – 1)
	District Hospital	3 (1 – 7)	2 (1 – 3)	NA	5 (3 – 10.5)
	Health Centre	7 (1 – 7)	1 (0.5 – 2)	NA	3 (2.5 – 6)
	Regional Hospital	2 (1 – 14)	1.5 (0.5 – 3)	NA	5.6 (2.5 – 12.1)
*Self-referral*		7 (2 – 30)	NA	2 (1 – 4)	2 (1 – 3.5)
Mode of transport					
Walking		NA	0.5 (0.17 – 4)	NA	15 (15 – 15)
Bicycle		NA	0.33 (0.33 – 0.33)	NA	NA
Motorcycle		NA	0.5 (0.5 – 1.25)	NA	NA
Car		NA	1.5 (1 – 2)	0.75 (0.33 – 36)	4.25 (0.75 – 5)
Public Transport		NA	2 (1 – 3)	2 (1 – 4)	5 (2 – 11.5)
Ambulance		NA	2 (2 – 2)	NA	3 (1.5 – 6.17)
*Caregiver demographics*					
Caregiver occupation	Private employee	3 (1 – 14)	1.5 (0.5 – 3)	2 (1 – 3)	4.375 (1.75 – 9)
	Public employee	2 (1 – 7)	1 (0.75 – 2)	2.5 (0.875 – 15)	4 (2 – 16)
	Homemaker	5.5 (1 – 21)	2 (0.75 – 3)	2 (2 – 2)	3.5 (2.33 – 14)
	Unemployed	2 (1 – 30)	0.35 (0.2 – 0.5)	10.75 (0.5 – 21)	4.5 (0.475 – 14.75)
Caregiver education	Higher Education	1.5 (1 – 7)	1 (0.5 – 2)	1 (0.5 – 10)	2.75 (1 – 12)
	Secondary	3.5 (1 – 21)	1.25 (0.5 – 2.75)	2.5 (1 – 12)	3.5 (2 – 8)
	Primary	2 (1 – 13)	1.5 (1 – 3)	2 (1 – 3)	4.5 (2 – 9)
	No education	4 (1 – 60)	3 (2 – 3)	NA	7 (4 – 17)
Age of caregiver	15–19	1 (1 – 30)	1 (0.5 – 3)	NA	7 (1.5 – 7)
	20–24	7 (2 – 14)	1 (0.5 – 1.5)	1 (1 – 2)	2.4 (1.5 – 3.75)
	25–29	3.5 (1 – 30)	1.75 (0.75 – 3)	2 (2 – 7)	4.4 (2 – 8.25)
	30–34	1 (1 – 4)	1.75 (0.5 – 4)	12 (1 – 18)	6 (2 – 16)
	35–39	1 (1 – 7)	2 (0.5 – 2)	2 (1 – 36)	3.1 (2 – 7)
	40–44	12 (1 – 35)	1.5 (0.5 – 3)	1 (0.75 – 3)	4.1 (1 – 10)
	> 45	7 (5.5 – 22)	0.5 (0.5 – 3)	0.75 (0.75 – 0.75)	10.4 (1.75 – 13)
Relationship status of caregiver	Married/cohabiting	3 (1 – 14)	1.5 (0.5 – 3)	2 (1 – 4)	4.25 (2 – 10)
	Single	2.5 (1 – 7)	0.75 (0.5 – 3)	NA	4,1 (1.75 – 9)
	Widowed/Separated	2.5 (1 – 30)	1.75 (0.5 – 2)	2 (1.165 – 7)	4 (2 – 5)
	Unknown	2 (2 – 2)	8 (8 – 8)	NA	18 (18 – 18)
Self-rated socioeconomic status	Enough money for food	4 (1 – 14)	1.75 (0.875 – 3)	1.5 (0.875 – 2)	4.25 (1.5 – 8.5)
	Enough money for food and clothes only	3 (1 – 10.5)	2 (0.5 – 3)	4 (3 – 12)	6.5 (3 – 12)
	Enough money for food, clothes, and savings	2 (1 – 30)	1 (0.5 – 2)	2 (1 – 7)	2.75 (1.46 – 9.5)
	Enough money for the above and certain expensive goods	1 (1 – 4)	0.6 (0.2 – 1)	0.75 (0.75 – 0.75)	0.75 (0.45 – 16)

For columns A), B), and C), the modes of transport being considered were those taken from the patient’s home. For column D), the modes of transport being considered were those taken from the patient’s home to MNH for self-referrals, and those taken from the referring health facility to MNH for referrals from health facilities
